# Psychometric properties of the German version of the Depressive and Anxious Avoidance in Prolonged Grief Questionnaire (DAAPGQ)

**DOI:** 10.1371/journal.pone.0254959

**Published:** 2021-08-10

**Authors:** Julia Treml, Michaela Nagl, Elmar Braehler, Paul A. Boelen, Anette Kersting

**Affiliations:** 1 Department of Psychosomatic Medicine and Psychotherapy, University of Leipzig, Leipzig, Germany; 2 Department of Medical Psychology and Medical Sociology, University of Leipzig, Leipzig, Germany; 3 Department of Psychosomatic Medicine and Psychotherapy, University Medical Center of the Johannes Gutenberg University Mainz, Mainz, Germany; 4 Department of Clinical Psychology, Utrecht University, Utrecht, The Netherlands; 5 ARQ National Psychotrauma Centre, Diemen, The Netherlands; Universitat d’Alacante, SPAIN

## Abstract

The *Depressive and Anxious Avoidance in Prolonged Grief Questionnaire* (DAAPGQ) was developed to measure depressive and anxious avoidance behaviors, which, according to cognitive-behavioral models, are supposed to play an important role in the development and maintenance of prolonged grief. The present study aimed to develop a German version of the DAAPGQ and evaluate its psychometric properties and validity within a representative sample of the German general population (N = 2531). The German-language DAAPGQ was developed using a forward-backward translation procedure. Then, a subsample of individuals who reported having lost a significant other (N = 1371) of a representative sample was assessed with the German DAAPGQ, along with information on sociodemographic characteristics, prolonged grief symptom severity (PG-13), general anxiety (GAD-2) and depression (PHQ-2). The factor structure of the DAAPGQ was evaluated using confirmatory factor analyses, reliability by calculating internal consistency on subscale level and convergent validity by correlations between DAAPQG subscale sores with PG-13, GAD-2 and PHQ-2 sum scores. As expected, a two-factor model with correlated latent variables showed good fit to the data, replicating findings from the original version. Internal consistency was high for both subscales (Cronbach’s α .86 and .95, respectively). Convergent validity was established by theoretically expected and statistically significant positive correlations of DAAPGQ subscales with symptom severity of prolonged grief, depression, and anxiety and negative correlations with time since loss. Furthermore, the addition of depressive and anxious avoidance significantly improved the prediction of prolonged grief symptom severity over sociodemographic and loss-related information. In sum, our results suggest that the German-language DAAPGQ is a reliable and valid measure of depressive and anxious avoidance and a useful tool to improve our knowledge on the role of avoidance in prolonged grief. We also provide descriptive data to improve the applicability of the DAAPGQ for individual diagnostics.

## Introduction

Grief is a natural response to the death of a loved one, and most bereaved are able to cope with the loss over time. For an estimated 10% of the bereaved, however, the grief reaction becomes abnormally persistent and causes significant impairment in functioning [1]. Considering only people bereaved by unnatural losses such as homicide, suicide or accidents, revealed an even substantially higher rate of 49% [2]. This condition, called Prolonged Grief Disorder (PGD), is part of the newest revision of the International Classification of Diseases (ICD-11) as a new diagnostic entity. The core symptoms are pervasive longing or yearning for the deceased or persistent preoccupation with the deceased coupled with functional impairment that extends beyond six months. Additionally, the American Psychiatric Association (APA) has now approved to include PGD in the forthcoming DSM-5-TR [3]. As the criteria for PGD changed over time, we use the term *PGD symptoms* throughout the manuscript to refer to the various grief disorders proposed over the years such as pathological, complicated, traumatic or prolonged grief or persistent complex bereavement disorder [4–8].

A Cognitive-behavioral theory proposes that PGD symptoms develop and persist due to three processes: (1) difficulties revising internalized representations of the self and the lost person to integrate the reality of the loss into the autobiographical memory (2) persistent negative global beliefs and misinterpretations of grief reactions, and (3) anxious and depressive avoidance behavior [9]. Therefore, cognitive-behavioral treatments concentrate on confronting the implications of the loss, changing negative cognitions, and reducing avoidance behaviors. These interventions have been shown to be effective for patients with PGD symptoms [e.g., 10–12].

With regard to avoidance behavior, a distinction can be drawn between depressive and anxious avoidance. The first refers to the avoidance of activities that could foster adjustment, such as social, recreational, and occupational activities. The depressive avoidance is driven by the view that these activities are useless and unfulfilling without the deceased. The latter refers to avoidance of stimuli associated with the loss due to the fear that confrontation with these reminders will be unbearable [13].

Although avoidance behavior is considered a central component of the cognitive-behavioral theory of PGD symptoms, its role in the development and maintenance of PGD symptoms is not well understood. To advance the understanding of underlying mechanisms of PGD symptoms and investigate the role of avoidance after bereavement, Boelen and Van Den Bout [14] developed the *Depressive and Anxious Avoidance in Prolonged Grief Questionnaire* (DAAPGQ). Nine items were generated, five tapping depressive avoidance (DA) and four tapping anxious avoidance (AA). All items were developed based on literature on coping with loss as well as interviews with bereaved suffering from “emotional complications after their loss” [14]. The initial validation of the questionnaire provided evidence that both forms of avoidance are distinct and that they contribute to the explained variance of symptom-levels of PGD, even after controlling for demographic and loss-related variables. Furthermore, the study showed that DA and AA were inversely related to time since loss. However, the correlations were low, suggesting that avoidance tendencies only marginally decline over time once they are present [14].

The inclusion of the DAAPGQ into observational studies has yielded some interesting insights into the role of depressive and anxious avoidance in PGD symptoms. Boelen and Eisma [13] found that both depressive and anxious avoidance are correlates of PGD symptoms and that anxious avoidance in the first year of bereavement is a prospective risk factor for PGD symptoms. Depressive avoidance was also found to be a significant mediator of the association between violent loss and PGD symptoms, depression, and posttraumatic stress disorder (PTSD), whereas anxious avoidance was a mediator of the association between violent loss and PTSD [15].

A study investigating the role of negative cognitions and avoidance behavior in PGD symptoms, PTSD, anger, and revenge thoughts after homicide revealed that those who engage in depressive avoidance showed higher levels of PGD symptoms and PTSD, whereas anxious avoidance was associated with elevated revenge thoughts and feelings [16]. Additionally, a recent study revealed that depressive and anxious avoidance partially mediated the associations between PGD symptoms and worrying and rumination, suggesting that targeting both depressive and anxious avoidance could reduce rumination [17]. These results complement the findings of Eisma and colleagues, who found that avoidance behaviors mediated the link between grief rumination and PGD symptoms as well as depression [18].

Taken together, these results underline the assumption that avoidance behavior plays an important role in the development and persistence of PGD symptoms and other emotional distress after losing a loved one. However, all these studies were conducted in the Netherlands, and more international research is necessary. As a precondition for this goal, the present study aimed to develop a German version of the DAAPGQ, examine its psychometric properties and evaluate validity.

The original DAAPGQ was first applied in the context of an observational study in which the validation of the instrument was not the actual goal. The sample was therefore small. Nonetheless, the findings demonstrated factorial validity as well as convergent validity [14]. An empirically validated measure of grief-related avoidance is not only essential to support research. Such an instrument can also help clinicians identify patients at risk of developing PGD and assist therapists in individualized treatment planning and monitoring treatment progress.

The inclusion of PGD in the ICD-11 and the forthcoming DSM-5-TR further emphasizes the need to assess etiological factors that contribute to this disorder, such as grief-related avoidance. Moreover, since PGD must be explicitly considered with particular attention to cultural differences in the duration of symptoms and grief expression (Killikelly and Maercker, 2017), validated translations of questionnaires like the DAAPGQ are indispensable. We, therefore, aimed to provide a German translation of the DAAPGQ and extend the psychometric testing and validation. Concerning the factorial structure, we expected an acceptable model fit for a two-factor model comparable to the original version. Regarding convergent validity, we predicted the DAAPGQ would be positively associated with symptoms of PGD, depression, and anxiety and marginally decline under the influence of time and therefore show a small but negative correlation with time since loss. Associations between the DAAPGQ, PGD, depression and time since loss have already been investigated (as described above). The association between the DAAPGQ and anxiety, however, has not yet been examined. Given that PGD and anxiety symptoms have been shown to be low to moderately correlated [19–21], we would expect similar correlations between the DAAPGQ and anxiety. Anxiety and avoidance reinforce each other, which in turn can hinder the processing of grief-related information [22] and thus contribute to the development of PGD. We therefore expected at least low associations between depressive and anxious avoidance and anxiety. Additionally, we assumed that depressive and anxious avoidance would predict symptoms of PGD, even after controlling for sociodemographic and loss-related variables.

## Materials and methods

### Procedures

A representative sample of the German population was drawn to be included in a cross-sectional survey between November 2017 and February 2018 based on a three-stage random sampling procedure: 1) random selection of 258 regional sample point areas, 2) random selection of target households within sample point areas based on a random-route procedure, 3) random selection of target persons within target households based on a Kish selection grid. Inclusion criteria for target persons were age equal or above 14 years, sufficient fluency in German language, and written informed consent. Random sampling and fieldwork were conducted by an independent market and social research agency (USUMA, Berlin, Germany). Target persons were approached without an appointment by one of 223 trained interviewers, up to four attempts were made to reach a target person. Potential participants received oral and written information about the study and provided informed consent. For target persons under the age of 18, additional parental informed consent was obtained. Sociodemographic information was collected in a face-to-face interview. Afterwards, participants filled out self-report measures on their own, and interviewers assisted in case of questions. The study and the procedures were approved by the local ethical review board (Leipzig University, Medical Faculty; AZ: 418/17-ek, 23.10.2017).

### Participants

Of a total number of 5093 randomly selected target persons, 2531 (49.7%) could be included in the study. Reasons for non-response were that a) households could not be reached (n = 731, 14.4%), b) households refused to participate (n = 840, 16.5%), c) target persons could not be reached (n = 181, 3.6%), d) target persons refused to participate (n = 804, 15.8%). Six interviews were not applicable for analyses. Bias due to non-response was limited by household- (household size) and person-level (age, gender, place of residence according to federal state) weights. Of the 2531 individuals included in the study, 1371 (54.2%) reported having experienced the loss of a significant person.

### Measures

The constructs used for the present study and described below were part of a larger thematically diverse set of self-report questionnaires implemented in the cross-sectional survey.

#### Sociodemographic information

Characteristics of the participant (age, gender, and education level) and characteristics of the deceased and the loss (e.g., relationship to the deceased, cause of death, time since death) were measured with a self-constructed questionnaire.

#### Depressive and Anxious Avoidance in Prolonged Grief Disorder (DAAPGQ)

The DAAPGQ was developed to examine the role of depressive (DA) and anxious avoidance (AA) in PGD. The questionnaire contains nine items rated on an 8-point scale ranging from 1 = “not at all true for me” to 8 = “completely true for me.” Five items were constructed to measure DA (range 5–40) and four items to measure AA (range 4–32). The first investigation revealed internal consistencies of .90 for the DA subscale and .74 for the AA subscale and both subscales were correlated with r = .77. [14]. The German Version of the DAAPGQ is provided within the S1 File. Two psychologists (JT and MN) independently translated the English version of the DAAPGQ into German. Both versions were compared for differences and merged by consensus into one German version. This version was then back translated by a native speaker. The back-translated version was then discussed with the original author (PB) for equivalence.

#### Symptoms of prolonged grief disorder

Symptoms of PGD were assessed using the German version of the PG-13 [6]. The PG-13 contains 13 items, 11 items assessing cognitive, behavioral, and emotional symptoms, rated on a 5-point Likert scale, and two items on duration and impairment that are to be answered “yes” or “no”. The severity of PGD symptoms can be evaluated by summing the scores obtained from these 11 items, leading to a sum score range of 11–55. Higher scores indicate more severe grief symptoms. To meet the criteria for PGD symptoms according to Prigerson et al. [6], the participant had to experience separation distress (one out of two items scored with 4 or 5) and cognitive, emotional, and behavioral symptoms (five out of nine items scored with 4 or 5). Furthermore, the symptoms had to be elevated for at least six months (symptom duration ≥ six months), and the participant must have significant impairment in social, occupational, or other important areas of functioning [6]. The PG-13 has been demonstrated to be reliable and valid [23]. Cronbach’s alpha in the present study indicated excellent internal consistency for the PG-13 (α = .94).

#### Depressive and anxiety symptoms

The Patient Health Questionnaire-4 (PHQ-4) was used to assess core symptoms of depression (PHQ-2) and core symptoms of generalized anxiety disorder (GAD-2) with two items each. The four items are rated on a 4-point scale ranging from 0 (not at all) to 3 (nearly every day), leading to sum score ranges between 0 and 6 for both subscales. The PHQ-4 demonstrated good psychometric properties and serves as a screening instrument for depressive and anxiety disorders (PHQ-2 and GAD-2 ≥ 3) [24]. Higher scores indicate higher degrees of depressive and anxiety symptoms [25]. The internal consistencies of the PHQ-4, PHQ-2 and the GAD-2 were good to acceptable in the present study (α = 0.87; α = 0.79; α = 0.80, respectively).

### Statistical analysis

All statistical analyses were conducted using the Statistical Package for Social Sciences, version 25 (IBM® SPSS®), including the software Analysis of Moment Structures, version 25 (IBM® SPSS® Amos). The significance level was set to α = .05.

#### Item analyses

We examined the item descriptives, item difficulties (in %) using the formula p_i_ = ((${\bar{x}}_{i}$−min(x_i_))/(max(x_i_)-min(x_i_))*100 (with ${\bar{x}}_{i}$ = mean of item i; min(x_i_) = minimal value on item i; max(x_i_) = maximum value on item i), corrected item-total-correlations, and percentages of missing values.

#### Reliability

Scale reliability was determined via internal consistency. Internal consistency was calculated using Cronbach’s α with α ≥ .70 indicating acceptable reliability. Mean inter-item correlations were calculated as an indicator of subscale homogeneity.

#### Construct validity

*Factor analysis*. The internal factor structure of the DAAPGQ as an indicator of construct validity was assessed with confirmatory factor analysis (CFA) based on structure equation modeling using the software AMOS, version 25. We predicted a two-factor model with two correlated latent variables (DA and AA). As the χ2-test as a global measure for the model fit is largely influenced by sample size, the following close fit indices and corresponding cut-off criteria were additionally used to evaluate the model fit:(1) Root Mean Square Error of Approximation (RMSEA) including the 90% confidence interval, with values ≤ .05 indicating good model fit, values between .05 and .08 indicating an acceptable fit, (2) Standardized Root Mean Square Residual (SRMR), with values < .10 indicating acceptable and values < .05 indicating good model fit, and (3) Comparative Fit Index (CFI) and (4) Tucker-Lewis Index (TLI), with values > .95 indicating acceptable and ≥ .97 indicating good model fit [26, 27]. As the original model assuming two correlated factors and uncorrelated errors showed only partially acceptable fit to the data, minor modifications to the model were necessary. Therefore, the total sample was split into two random samples using the SPSS 25 random selection procedure. Model modifications, guided by an inspection of modification indices and only included if they were theoretically plausible, were performed in the first split-half sample and the final model was then cross-validated in the second split-half sample.

*Convergent validity*. Convergent validity was assessed as an indicator of construct validity by calculating Pearson’s correlation coefficients between subscales of the DAAPGQ and theoretically related constructs, i.e. PGD symptoms (PG-13) and depressive and anxiety symptoms (PHQ-4). We predicted both subscales DA and AA to be positively correlated with these measures. Furthermore, we predicted that DA and AA would marginally diminish over time and therefore negatively correlate with time since loss.

Additionally, a hierarchical regression analysis was performed to further establish construct validity. We expected that DA and AA would predict symptoms of PGD, even after controlling for sociodemographic variables, variables related to the loss, and the relationship to the deceased. Only participants who fulfilled the ICD-11 time criterion (time since loss ≥6 months) were included in this analysis. Four blocks of independent variables were entered hierarchically in order of their point in time related to the loss (pre-loss, loss, peri-loss): sociodemographic variables (age, gender); variables related to the loss (time since loss, number of losses, cause of death); relationship to the deceased; and DA and AA. Dummy variables were generated for the categorical variables cause of death and relationship to the deceased. Reference groups were anticipated natural death (e.g., illness) and losing a parent, respectively. The categories of accident, suicide, and violent death were grouped into sudden violent death. The variance inflation factor was calculated for the diagnosis of multicollinearity. Values above 5 were considered as an indication of multicollinearity [28].

## Results

### Participant and loss-related characteristics

A total of 1371 (54.2%) individuals reported having experienced a major bereavement. Table 1 gives an overview of the demographic characteristics of the bereaved sample. Loss-related characteristics are displayed in Table 2. Bereaved individuals were aged between 14 and 93 years (M = 54.56, SD = 17.28). About 59% were women, about 56% were living with a partner, and the great majority (97.1%) reported German nationality (Table 1). The time since the loss of the significant person ranged between 0 and 976 months (M = 128.14, SD = 138.11). About 40% indicated the time since the loss to be greater than 10 years. The majority (46.6%) reported to have lost a parent, and sudden natural death was reported most frequently (44.7%) as the cause of death (Table 2). According to the diagnostic algorithm of the PG-13, 3.3% (n = 45) met the criteria for clinically relevant PGD symptoms. Those with relevant PGD symptoms were mostly female (71%; n = 32), mean age was 61.09 (SD = 18.80), mean time since loss was 69.91 months (range: 7–456 months, SD = 100.31). About half of them reported to have lost a partner (n = 23), and a quarter lost a parent (n = 11), 9% lost their child (n = 4). The most frequently indicated cause of death was a disease (n = 21), followed by sudden natural deaths (n = 16).

**Table 1:** Demographic characteristics of the bereaved sample.

		Bereaved sample (n = 1371)	
		N	%
Gender	Male	562	41.0
	Female	809	59.0
Age, years	≤ 24	81	5.9
	25–34	133	9.7
	35–44	158	11.5
	45–54	261	19.0
	55–64	295	21.5
	65–74	273	19.9
	≥ 75	170	12.4
Nationality	German	1331	97.1
	Other	40	2.9
Education	< 12 years	1072	78.5
	≥12 years	279	20.4
	Attending school	15	1.1
Employment status	Employed	1307	96.1
	Unemployed	53	3.9
Marital status	Married	654	47.9
	Single	310	22.7
	Divorced	191	14.0
	Widowed	209	15.3
Living with a partner	Yes	762	56.0
	No	599	44.0
Monthly household net income	< 1250 EUR	214	16.3
	1250–2500 EUR	588	44.7
	≥ 2500 EUR	512	39.0

**Note.** Percentages were calculated from valid cases.

**Table 2:** Loss-related characteristics of the bereaved sample.

		Bereaved sample (n = 1371)		
		M	SD	Range
Age at the time of the loss, years		43.69	18.45	0–88
Time since loss, months		128.14	138.11	0–976
Age of the deceased, years		66.79	19.04	0–98
		N	%	
Time since loss	< 6 months	48	3.5	
	6–12 months	57	4.2	
	1–2 years	123	9.1	
	2–5 years	315	23.2	
	5–10 years	275	20.3	
	> 10 years	537	39.6	
Gender of the deceased	Male	632	47.9	
	Female	688	52.1	
Relationship to the deceased	Spouse	204	15,2	
	Child	32	2.4	
	Parent	624	46.6	
	Sibling	66	4.9	
	Grandparent	217	16.2	
	Parent in law	16	1.2	
	friend	95	7.1	
	Other	86	6.4	
Cause of death	Sudden natural death	610	44.7	
	Disease	528	38.7	
	Accident	101	7.4	
	Suicide	35	2.6	
	Violent death	6	0.4	
	Other causes	84	6.2	

**Note.** Means and percentages were calculated from valid cases.

### Item analyses

The item characteristics of the German version of the DAAPGQ are displayed in Table 3. Item means on the eight-point response scale (range 1–8) ranged from 1.79 (item 3) to 3.01 (item 9). All items were positively skewed. Skewness ranged from 0.76 (item 9) to 2.22 (item 3). Seven items showed a positive and two items (8, 9) a negative kurtosis. Kurtosis ranged from -0.70 (item 9) to 4.39 (item 3). Item difficulties ranged between 13.19% (item 3) and 33.51% (item 9), indicating a low to medium probability of scores > 1 (‘not at all true for me‘). Corrected item-total-correlations were high (0.60 ≤ *r*_it_ ≤ 0.79), indicating an adequate discriminatory power. The percentage of missing values was low (≤ 0.95%) for all items.

**Table 3:** Psychometric properties of the DAAPGQ items.

	M	SD	Skew	Kurt	p_i_ (%)	r_it_	% missings
**Depressive Avoidance**							
1. Since [–] is dead, I do much less of the things that I used to enjoy.	2.04	1.74	1.78	2.31	17.36	0.90	0.58
2. Since [–] died, I avoid activities that used to give me satisfaction, because these activities now seem meaningless to me.	1.89	1.59	1.99	3.38	14.89	0.91	0.66
3. I avoid doing activities that used to bring me pleasure, because I feel unable to carry out these activities.	1.79	1.53	2.22	4.39	13.19	0.90	0.73
4. I develop very few new activities since [–] died, because I am unable to do so.	1.84	1.61	2.16	4.04	14.06	0.89	0.80
5. Since [–] died, there are several activities, hobby’s, and acquaintances that I pay much less attention to.	2.10	1.80	1.71	2.04	18.32	0.78	0.73
**Anxious Avoidance**							
6. I avoid to dwell on the fact that [–] is dead and will never return.	2.70	2.16	1.11	0.02	28.38	0.73	0.95
7. I avoid situations and places that confront me with the fact that [–] is dead and will never return.	2.26	1.86	1.54	1.42	20.92	0.69	0.88
8. I avoid to dwell on painful thoughts and memories connected to his/her death.	2.76	2.13	1.01	-0.18	29.34	0.79	0.66
9. I deliberately retrieve positive memories related to [–] as a means to avoid thinking about the fact that [–] is dead and will never return.	3.01	2.20	0.76	-0.70	33.51	0.64	0.95

M = mean, SD = standard deviation, skew = skewness, kurt = kurtosis, p_i_ = item difficulty, r_it_ = corrected item-total correlation on subscale level

### Internal factor structure

CFAs were performed for cases with complete data in the DAAPGQ, resulting in a total sample of N = 1345. The original model assuming a two-factor model with uncorrelated errors in the total sample showed contradictory findings. While some indices indicated an acceptable fit to the data (CFI = .970, TLI = .958; SRMR = .057) the inspection of the RMSEA (RMSEA = .096, 90%CI: .087,.105) and the χ2-test (χ2 = 349.401, p > .001) indicated poor model fit. Therefore, minor model modifications were performed in the first split-half sample (n = 672). The final model, allowing for correlations of unique variances between two items from the depressive avoidances scale (items 3 and 4, items 4 and 5) as well as between two items from the anxious avoidance scale (items 6 and 8, items 7 and 9), showed a good fit to the data in the first split-half sample (χ2 = 95.529, p < .001; CFI = .987; TLI = .978; RMSEA = .071, 90%CI: .056,.085; SRMR = .023). These correlations were considered theoretically plausible due to similarities of the wording in these items and the overlaps in content, which may display a non-random measurement error. The cross-validation in the second split-half sample (N = 673) confirmed the good fit to the data (χ2 = 101.601, p < .001; CFI = .985; TLI = .976; RMSEA = .073, 90%CI: .059,.088; SRMR = .034). Because the χ2-test is strongly influenced by sample size, we did not take it into account as a global measure of model fit.

### Descriptive data and reliability

Table 4 presents the descriptive data for both subscales for the total sample and participants with and without PGD symptoms, according to the diagnostic algorithm of the PG-13. In the total sample, both subscales were positively skewed. The kurtosis for DA was also positive, for AA slightly negative. Cronbach’s α was .95 for DA, and .86 for AA.

**Table 4:** Descriptive data of the DAAPGQ subscales.

	M	SD	Range	Skew	Kurt
**Total sample** (N = 1359)					
Depressive Avoidance	9.68	7.60	5–40	1.93	3.13
Anxious Avoidance	10.71	7.02	4–32	0.92	-0.05
**Participants without PGD symptoms** (N = 1314)					
Depressive Avoidance	9.01	6.65	5–40	2.01	3.61
Anxious Avoidance	10.41	6.84	4–32	.96	.05
**Participants with PGD symptoms** (N = 45)					
Depressive Avoidance	29.20	8.02	10–40	-.35	-.86
Anxious Avoidance	19.60	6.47	8–32	.24	-.57

M = mean, SD = standard deviation, skew = skewness, kurt = kurtosis

**Note.** Means were calculated from valid cases.

Mean inter-item correlations were .81 for DA and .61 for AA. A significant correlation between the two subscales was found, with r = .55 (p < .001).

### Convergent validity

As expected, the subscales of the DAAPGQ were significantly correlated with symptoms of prolonged grief, depression, and anxiety and negatively with time since loss. All correlations are presented in Table 5. The highest positive correlation was found between the DA subscale and the PG-13 with r = .739, the lowest between AA and time since loss (r = -.204).

**Table 5:** Bivariate correlations between subscales of the DAAPGQ and theoretically related constructs.

	PG-13	PHQ-2	GAD-2	Time since loss sin
Depressive Avoidance	.739**	.420**	.318**	-.205**
Anxious Avoidance	.540**	.293**	.251**	-.204**

** p < .001

Furthermore, the results of the regression analysis demonstrate that DA and AA predict symptoms of PGD, even after controlling for sociodemographic variables, variables related to the loss, and the relationship to the deceased. The results are summarized in Table 6. The sociodemographic variables in step 1 explained 2.9% of the variance. Entering variables related to the loss (step 2) increased the explained variance to 9.6%. The third step (relationship to the deceased) led to an explained variance of 19.6%. DA and AA were entered in the last step, improving the model with changes in R^2^ of 39.8%. The final model explained 59.4% of variance (F (11,1156) = 153.744, p < .001), which can be considered a large effect size, according to Cohen [29].

**Table 6:** Results of hierarchical regression for predictors of PGD symptoms (n = 1168).

	Step 1			Step 2			Step 3			Step 4		
	Sociodemographic variables			Variables related to the loss			Relationship			Depressive and Anxious Avoidance		
	B	ß	p value	B	ß	p value	B	ß	p value	B	ß	p value
*Age*	**0.058**	**0.108**	**< .001**	**0.089**	**0.166**	**< .001**	**-0.018**	**-0.033**	**.330**	**-0.028**	**-0.051**	**.035**
*Gender*	**2.536**	**0.137**	**< .001**	**2.829**	**0.153**	**< .001**	**1.823**	**0.098**	**< .001**	**1.447**	**0.078**	**< .001**
*Number of losses*				0.047	0.013	.660	0.087	0.024	.392	0.053	0.015	.459
*Time since death*				**-0.017**	**-0.257**	**< .001**	**-0.013**	**-0.187**	**< .001**	**-0.003**	**-0.043**	**.039**
Cause of death (vs expected natural)												
Sudden natural				**-1.259**	**-0.069**	**.020**	-0.949	-0.052	.064	**-0.781**	**-0.043**	**.032**
Sudden violent				1.101	0.038	.209	-0.101	0.003	.905	-1.061	-0.036	.080
Relationship to the deceased (vs parent)												
Spouse							**8.216**	**0.324**	**< .001**	0.400	0.016	.507
Child							**10.395**	**0.171**	**< .001**	**4.494**	**0.074**	**< .001**
Other relatives							-0.946	-0.049	.117	-0.842	-0.044	.050
Depressive Avoidance										**0.751**	**0.614**	**< .001**
Anxious Avoidance										**0.238**	**0.181**	**< .001**
*R*^*2*^ (adjusted *R*^*2*^)	.029 (0.27)			.096 (.091)			.196 (.190)			.594 (.590)		
Δ*R*^*2*^	.029**			.067**			.101**			.398**		

B unstandardized regression coefficient; ß standardized coefficient; ΔR^2^ change in R^2^ induced by predictors entered at regression step

**p < .01

*p < .05

All VIF values were below 5, which means there was no indication of multicollinearity. The standardized regression coefficient showed that DA and AA provided a significant contribution to symptoms of PGD. More DA (ß = .61, p < .001), and more AA (ß = .18, p < .001) were associated with higher levels of prolonged grief symptoms.

## Discussion

The DAAPGQ measures avoidance behavior after the loss of a loved one and consists of the subscales depressive and anxious avoidance. This study aimed to provide a German version of the DAAPGQ and evaluate its psychometric properties and validity in a large sample of bereaved individuals drawn from a representative sample of the German general population. We also provide descriptive data on DAAPGQ scales which may help users of the DAAPGQ interpret their findings in relation to this normative representative sample.

The percentage of missing values in DAAPGQ items was very low (≤ 0.95%), indicating that the items were very well accepted by the study participants. Item descriptives showed that item distributions were positively skewed for both subscales, and corrected item-total correlations were high. Item difficulties were medium to high (range: 13.19–33.51%). Especially the items tapping depressive avoidance were rather difficult. When interpreting this fact, it is important to keep in mind that the questionnaire focuses on DA and AA in PGD and that both are correlates of PGD symptoms [14]. It is therefore plausible that some items are more relevant to a minority of bereaved experiencing a more persisting and disabling grief reaction. This is illustrated by our findings that participants with PGD symptoms reported much higher levels of DA and AA than participants without PGD symptoms. The more difficult items may have discriminatory power between people with and without PGD symptoms. In addition, the internal consistencies of the subscales of the DAAPGQ were good to excellent in this study and comparable to internal consistencies found for the original version in other studies [e.g., 15, 16].

With regard to the factorial validity and internal factor structure of the DAAPGQ, our study replicated the findings by Boelen and Van Den Bout [14], supporting a two-factor model with two distinct but correlated factors. To improve the model fit, we allowed for correlated error-terms for two items of each subscale. We consider these correlations theoretically plausible due to the overlaps in content and similarities of the wording in these items, which may display a non-random measurement error. This result supports the distinction of depressive and anxious avoidance in the cognitive-behavioral model of PGD symptoms [9, 14].

The validity of the DAAPGQ was further supported by this study. As expected, the subscales DA and AA only slightly diminished over time, as evidenced by the negative but mild correlation with time since the loss. This indicates that avoidance tendencies once manifested remain somewhat stable over time, which may contribute to the development of PGD. This result also replicates the findings by Boelen and Van Den Bout [14].

Additionally, both DA and AA were significantly and positively correlated to symptoms of PGD, depression, and anxiety. This is consistent with previous research and theoretical considerations that regard avoidance as a mechanism that fosters and maintains PGD symptoms [9]. Furthermore, PGD symptoms, in turn, are also associated with anxiety and depression [30]. Since DA and AA are thought to contribute to the development of PGD, we hypothesized that both should also correlate with depression and anxiety. Furthermore, avoidance behavior has been found a key mechanism in the maintenance of both depression and anxiety [31, 32].

The results of the regression analysis further demonstrated that both DA and AA predict symptoms of PGD. This association remains, even after controlling for sociodemographic variables, variables related to the loss and the relationship to the deceased. DA and AA explain almost 40% of the variance, which is a large proportion, underlining the importance of avoidance as an independent mechanism. All these results provide evidence for the validity of the DAAPGQ.

A major strength of this study is the large representative sample of the German general population, which extends the findings from Boelen and Van Den Bout [14] to a representative sample. This allows for the provision of normative descriptive data, which may help future users of the DAAPGQ interpret their findings in relation to our data and improve the applicability of the DAAPGQ with regard to individual diagnostics.

Major limitations are the cross-sectional design and the lack of data on retest-reliability and sensitivity to change, which is of particular importance when using the DAAPGQ in intervention studies. Furthermore, only a limited number of constructs were used to calculate construct validity, and no other measures of loss-related avoidance or general avoidance such as the Acceptance and Action Questionnaire-II [AAQ-II, 33] were applied to evaluate convergent validity. Lastly, we used the PG-13 to assess PGD symptoms. Even though it is one of the most widely used and well-established instruments for assessing PGD symptoms, its items do not assess all of the current PGD criteria included in the ICD-11 (nor the DSM-5-TR). At the time of assessment, there was no tool available capturing all the PGD ICD-11 criteria [23]. Thus, we recommend future research to assess PGD according to the latest ICD-11 criteria to provide more accurate normative, descriptive data of the DAAPGQ.

### Implications and conclusion

Despite these limitations, we can conclude that the DAAPGQ demonstrated good psychometric properties, and factorial and construct validity were established. Thus, the DAAPGQ can be considered a reliable and valid tool to assess grief-related avoidance after losing a loved one. We, therefore, recommend the use of the German version in German-speaking countries. As prior study results suggest, reducing DA and AA should be central within psychological interventions for persistent distress following the loss of a loved one [14, 15]. Targeted interventions adapted to the needs of the bereaved might be most efficacious. Thus, more knowledge on the isolated effectiveness of interventions targeting DA and AA might be beneficial. There is already preliminary evidence that interventions targeting DA are effective [34]. The DAAPGQ could also serve as a tool to assess treatment progress by monitoring the scores during the course of treatment. For this, however, the sensitivity to change of the DAAPGQ should be investigated first, which we recommend for future research. As the DAAPGQ is short with only nine items, it should be easy to implement within efficacy or effectiveness research of grief therapy.

## Supporting information

S1 File (DOCX)

S1 Table (XLSX)
