# Supplementary material for: Psychometric properties of the German version of the Depressive and Anxious Avoidance in Prolonged Grief Questionnaire (DAAPGQ)
Source: PLoS One. 2021 Aug 10;16(8):e0254959. doi: 10.1371/journal.pone.0254959 (PMC8354469; doi:10.1371/journal.pone.0254959)
Supplement: S1 File — (DOCX) [file pone.0254959.s001.docx]

**Fragebogen zur depressiven und ängstlichen Vermeidung bei prolongierter Trauer (DAAPGQ)**


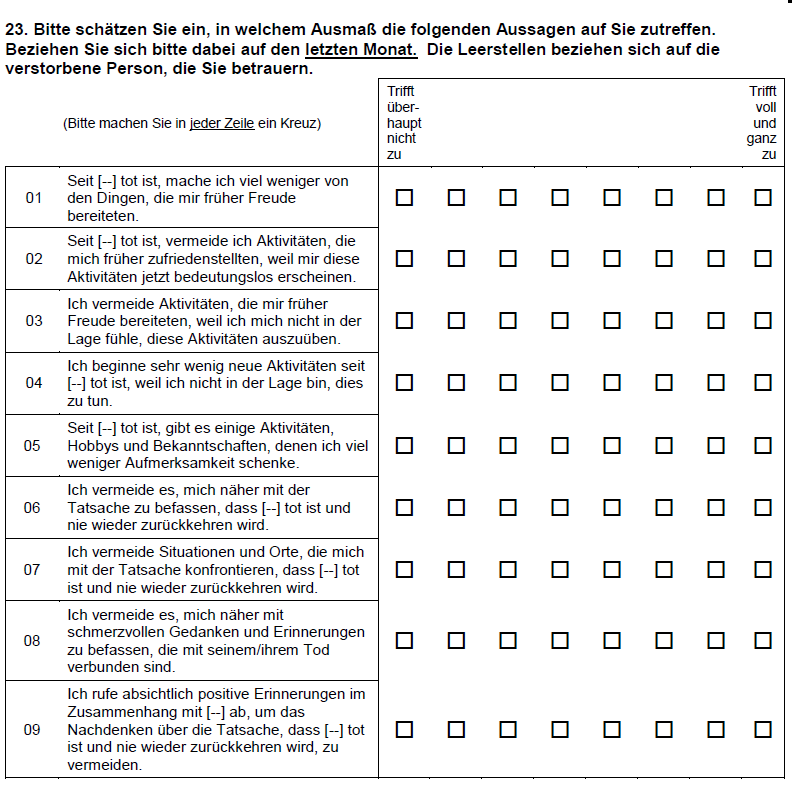


Deutsche Übersetzung: Treml, Nagl, Kersting & Boelen
